# De-Escalating Breast Cancer Surgery: Should We Apply Quality Indicators from Other Jurisdictions in Canada?

**DOI:** 10.3390/curroncol29010013

**Published:** 2021-12-29

**Authors:** Hannah Kapur, Leo Chen, Rebecca Warburton, Jin-Si Pao, Carol Dingee, Urve Kuusk, Amy Bazzarelli, Elaine McKevitt

**Affiliations:** 1 Providence Breast Centre, Mount Saint Joseph Hospital, 3080 Prince Edward Street, Vancouver, BC V5T 3N4, Canada; hannah.kapur@alumni.ubc.ca (H.K.); RWarburton@providencehealth.bc.ca (R.W.); JPao@providencehealth.bc.ca (J.-S.P.); Ckdingee@telus.net (C.D.); urve.kuusk@ubc.ca (U.K.); ABazzarelli@providencehealth.bc.ca (A.B.); 2 Department of Surgery, Faculty of Medicine, University of British Columbia, 2775 Laurel Street, Vancouver, BC V5Z 1M9, Canada; leochen1@mail.ubc.ca

**Keywords:** breast neoplasms, mastectomy, segmental, mastectomy, quality indicators, health care

## Abstract

Quality Indicators (QIs), including the breast-conserving surgery (BCS) rate, were published by the European and American Breast Cancer Societies and this study assesses these in a Canadian population to look for opportunities to de-escalate surgery. A total of 2311 patients having surgery for unilateral, unifocal breast cancer between 2013 and 2017 were identified and BCS QIs calculated. Reasons for mastectomy had been prospectively collected with synoptic operative reporting. Our BCS rate for invasive cancer < 3 cm was 77.1%, invasive cancer < 2 cm was 84.1%, and DCIS < 2 cm was 84.9%. There was no statistically significant change in BCS rates over a five-year period, but there was a reduction in contralateral prophylactic mastectomies (CPM) from 28% in 2013 to 16% in 2017 (*p* < 0.001). Trend analysis looking at tumour size and medical need for mastectomy indicated that 80% of patients at our centre would be eligible for BCS with tumour cut off of 2.5 cm. Our institution met American but not European QI standards for BCS rates, potentially indicating a difference in patient demographics compared to Europe. Our results support the understanding that BCS rates are influenced by multiple factors and are challenging to compare across jurisdictions. CPM rates may offer a more actionable opportunity to de-escalate surgery for breast cancer.

## 1. Introduction

Breast cancer is the most common cancer in Canadian women and one in eight women are expected to develop breast cancer in their lifetime [1]. Improved survival has been observed with early detection and tailored treatment. Surgery is a critical first-line treatment for early-stage breast cancer. Before the 1980s, there was controversy over the surgical management of breast cancer between total mastectomy (TM) or breast-conserving surgery (BCS). Landmark randomised control clinical trials established the safety of BCS for treatment of Stage I or II breast cancer, finding that BCS plus radiation had equivalent survival compared to TM [2,3]. These findings led to BCS plus radiation being recommended for early-stage breast cancer, assuming that clear margins and an acceptable cosmetic outcome could be obtained [4]. Hence, BCS presents an opportunity to de-escalate surgical treatment.

Despite multiple randomised studies showing the safety of BCS and more recent observational studies [5] suggesting improved outcomes over mastectomy, the rates of mastectomy are still high. In fact, mastectomy rates are increasing and there is even a trend towards requesting contralateral prophylactic mastectomies (CPM) [6,7,8,9]. The literature has identified many factors in the patient decision-making process that leads patients to choose more radical surgery, including the perception of survival advantage, fear of recurrence, and desire to avoid radiation or distance to radiation centres [10,11,12,13,14,15]. Some patients also prefer CPM in order to achieve symmetry or better reconstruction results [10,11,12,13,14,15]. Many of these fears and factors can be addressed by patient education and strong therapeutic relationships.

The European Society of Breast Cancer Specialists (EUSOMA) and the American College of Surgeons National Accreditation Program for Breast Centres (ACS-NAPBC) have published manuals of Quality Indicators (QIs) to be measured and health care standards to be met at breast centres [16,17,18,19,20]. Clinicians in Europe have already investigated the feasibility of calculating QIs using clinical and administrative data, and while North American centres are beginning to follow suit, there are currently no surgical breast cancer QIs published for Canadian centres. One recommended QI is the BCS rate, the goal of which is to prevent overtreatment of patients that are eligible for BCS treatment by minimising mastectomy rates. In 2012, our institution, the Providence Health Care Breast Centre, reviewed our practice and we found that we met all ACS-NAPBC and EUSOMA QIs, however, we found lower than expected BCS rates, at our institution.

The finding of higher than expected mastectomy rates led to the present study. Our primary purpose was to calculate QIs for BCS rate and determine compliance with American and European standards between 2013 and 2017. Our secondary purpose was to examine reasons for mastectomy and identify opportunities to de-escalate surgery. We hypothesised that mastectomy rates are higher at our institution than European standards due to the number of medically necessary mastectomies.

## 2. Materials and Methods

### 2.1. The Clinical Data

**Figure 1:**
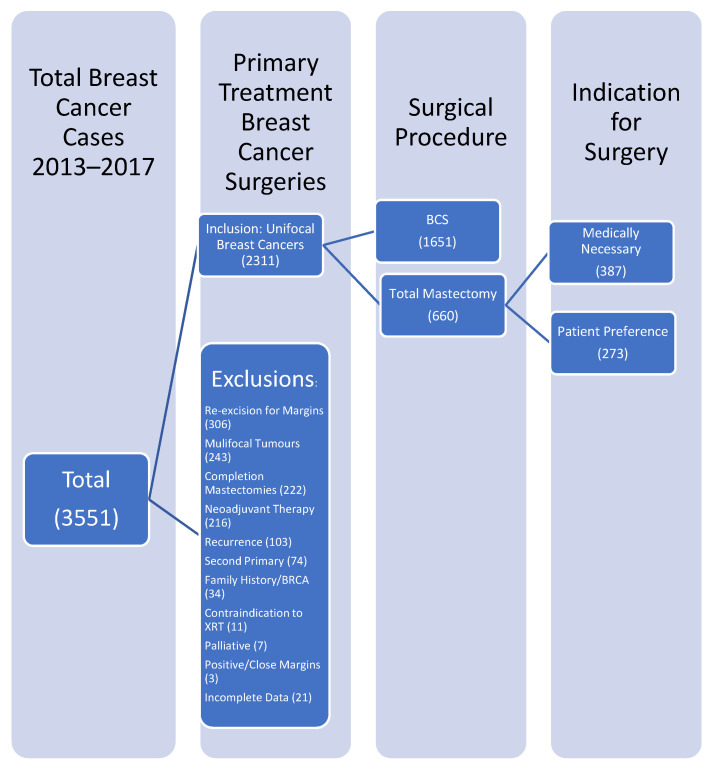
Overview of breast cancer surgeries performed.

This study was approved by the University of British Columbia Research Ethics Board. The Providence Breast Centre was established in 2009 and prospectively tracks surgical procedures for quality improvement. Surgeons prospectively record indications for surgery, preoperative tumour characteristics, and reason for mastectomy and axillary dissection using our provincial synoptic operative report. The multidisciplinary team includes surgical oncology and breast reconstruction surgeons. We identified all patients who received breast cancer surgery, BCS or TM, between 2013 and 2017 in our surgical database. Patients with unifocal first diagnosis of breast cancer having upfront surgery were included. Patients with multifocal disease, neoadjuvant therapy, contraindication to radiotherapy, BRCA1/2 predispositions and second surgical procedures (margin re-excision, completion mastectomy) were excluded (Figure 1). Between 2013 and 2017, 3551 breast cancer procedures were performed and 2311 met inclusion criteria. The “reason for mastectomy” for each patient undergoing TM was prospectively collected using our provincial synoptic operative report and verified by chart review to delineate patients receiving a mastectomy for medical reasons, such as “tumour too large for size of breast”, or patient preference. Patients undergoing TM were divided into TMs that were medically necessary (TMMN) and TMs that were by patient preference (TMPP). Pre-operative size was the largest of clinical exam, mammogram, ultrasound, and MRI. The imaging tests performed were at the discretion of the radiologist/surgeon and MRI was used selectively. Post-operative size was pathological size of the invasive component when invasive disease was present as the DCIS component was not consistently measured when invasive disease was present.

### 2.2. Outcome Measures

QIs relating to surgical breast procedures were identified from the European Society of Breast Cancer Specialists (EUSOMA) [17] and the American College of Surgeons National Accreditation Program for Breast Centres (ACS-NAPBC) [18]. We were interested in evaluating against the 2017 update of EUSOMA QIs 9a, 9b, 9c, 11c, and 11d. We also evaluated against NAPBC Standard 2.3. Outcome measures were compared for compliance to the standards set by EUSOMA and ACS-NAPBC.

### 2.3. Statistical Analysis

All statistical analyses were carried out using R version 3.6.2. *p*-values smaller than 0.05 were considered statistically significant. Patient and tumour characteristics were compared across three groups using pair-wise analysis; BCS vs. TMMN, BCS vs. TMPP, and TMMN vs. TMPP. Patients with missing data were excluded. T-tests were used to compare numeric outcomes as means and Wilcoxon rank sum tests were used to evaluate numeric outcomes as medians. Categorical outcomes were compared using Chi-squared tests unless categories had fewer than five values in which case Fisher’s Exact tests were used. Odds ratios for QI time-trends and age-trends were evaluated using logistic regression. Relative rates for CPM time-trends were calculated using a Poisson regression model.

## 3. Results

Figure 1 describes the patient population studied.

Overall, 3551 breast cancer procedures were performed including 1447 mastectomies, which were 40.7% of breast cancer procedures performed at our regional centre. Of the 2311 patients that met inclusion criteria, 1651 patients underwent BCS and 660 underwent TM. This study population was used to calculate QIs. Within the TM study group, evaluation of prospectively-collected “reason for mastectomy” revealed that 387 were medically necessary (TMMN) and 273 were by patient choice (TMPP). The TMPP cases represent 7.7% of all breast procedures at our institution and 18.9% of our mastectomies performed. Overall, during the study period, 1174 (81.1%) mastectomies were performed that were classified as medically necessary. For patients with BCS at our centre in 2013–2016, 104 patients (8.1%) had a completion mastectomy within a year.

Patient and tumour characteristics are reported in Table 1.

There was a statistical difference in the median age of patients, with a higher proportion of younger patients receiving TMMN (*p* < 0.001). For CPM rates, the TMPP group had a significantly greater CPM rate than TMMN (*p* < 0.05). Patients receiving TM, either TMMN or TMPP, were more likely to have bilateral cancer than BCS (*p* < 0.001), although there was no statistical difference in bilateral cancer rates between TMMN and TMPP. The TMMN group had a statistically larger reconstruction rate than the TMPP group (*p* < 0.001). Tumour pre- and post-op sizes were significantly larger in the TMMN (*p* < 0.001). TMMN were also more likely to be lymph node-positive (*p* < 0.001). Patients in the TMMN were more likely to initially present with a mass, while patients in the TMPP and BCS groups were more likely to present with an imaging abnormality. Within the patient preference group, we did not appreciate a geographical influence when assessing postal codes to estimate patients travelling to our centre from outside our region (data not shown).

The six Qis being evaluated are listed in Table 2, which displays the description for each QI, the minimum standard, and the target rate.

Our institution’s BCS rate according to the APC-NAPBC standard was 71.4%, which meets the recommended target. The EUSOMA single-operation rate for invasive cancer was 88.8% and 80.3% for in situ cancer (DCIS), which both met the minimum standard but not the target. Our BCS rate for invasive cancer <3 cm was 77.1%, and DCIS < 2 cm was 84.9%, which also both met minimum standards but not targets.

We then removed the TMMNs from the QI calculations and compared for compliance (Table 2). The BCS rate according to the APC-NAPBC standard increased to 81.2%. The EUSOMA single operation rates decreased to 84.5% for invasive cancer and 69.6% for DCIS. The EUSOMA BCS rates increased to 83.4% for invasive cancer and 90.1% for DCIS, meeting targets.

Time and age trends for BCS rates by tumour type and size are reported in Table 3 using logistic regression analysis.

**Figure 2:**
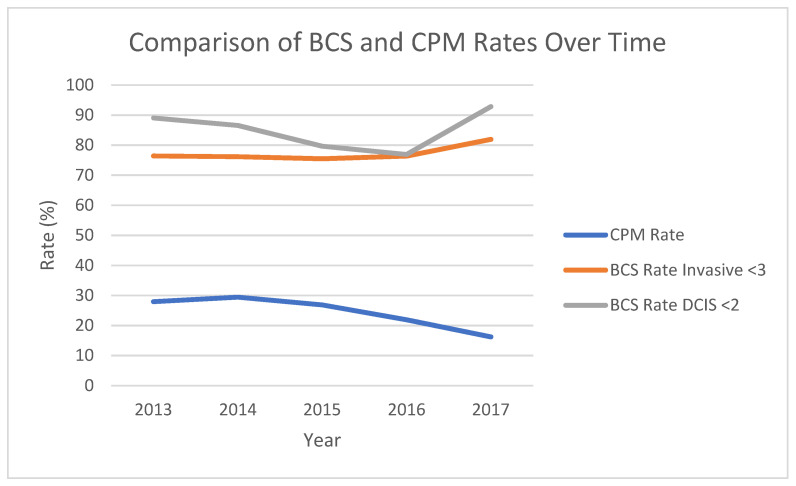
Time trends in BCS rate for invasive cancer, BCS rates for DCIS and CPM rates.

Over the five-year period, between 2013 and 2017, there were no statistically significant changes in BCS rates for invasive cancer <3 cm, invasive cancer <2 cm, or DCIS < 2 cm. The three age groups chosen for comparison were patients aged less than 40, 40 to 74, and 75 and above. Among patients with invasive tumours <3 cm, those between the ages of 40 and 74 had 2.54 odds for receiving BCS compared to those younger than 40, which was significant. Figure 2 graphically represents no change in BCS rates over time for DCIS and invasive disease.

However, there was a reduction in CPM rates over time at our institution, decreasing from 28% to 16% between 2013 and 2017 (95% CI 0.808–0.934, *p* < 0.001, RR 0.869).

**Figure 3:**
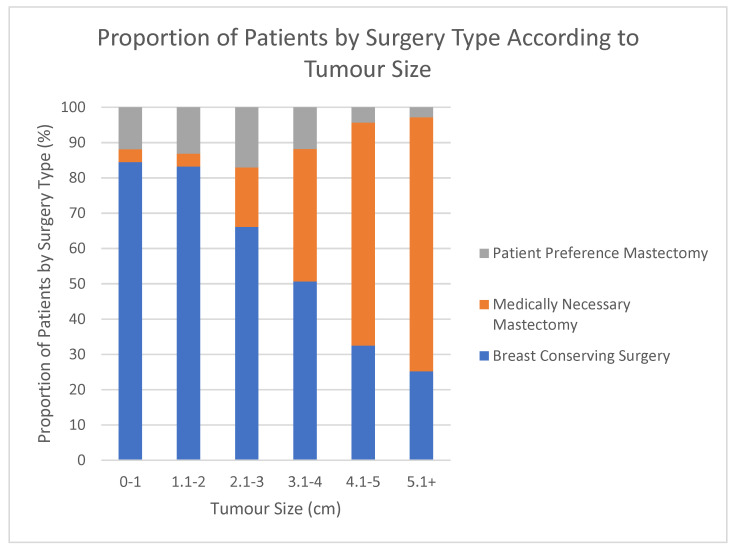
Proportion of patients with invasive cancer receiving either BCS, TMMN, or TMPP by tumour size.

Figure 3 represents patients with invasive cancer grouped by their tumour size in 1 cm increments.

Within each invasive tumour size, the proportion of patients receiving BCS, TMMN, and TMPP was indicated. Trend analysis revealed that 80% of our patients would be eligible for BCS with a preop tumour size cut-off of 2.5 cm inclusively. For patients with preop tumour size up to 2 cm in size 135 patients had mastectomies: 31 patients TMMN (2.8%) and 104 patients TMPP (9.7%).

## 4. Discussion

QIs for BCS rates are designed to capture the amount of breast cancer that may be overtreated with mastectomy. On the calculation of our institution’s five-year BCS rates between 2013 and 2017, our institution found that we met American QI targets (NAPBC 2.3) but not European (EUSOMA QI 11c and 11d) targets, indicating that mastectomy rates were higher at our institution than recommended in Europe. Unique to our study was prospectively collecting our patients’ “reason for mastectomy” in order to identify why mastectomies were performed at our institution. As carried out in previous studies [15], we identified that patients fell into medically necessary (TMMN) and patient preference (TMPP) groups. At our institution, we found that more patients had mastectomies for medically necessary reasons than by patient preference. On removal of the TMMN patient group from the QI calculations, our BCS rates improved and we reached European (EUSOMA 11d and 11c) QI targets. This finding supports our hypothesis that mastectomy rates at our institution are due to a higher number of medically necessary mastectomies. Furthermore, the removal of medically necessary mastectomies from the QI calculation better represented the number of mastectomies that could have reasonably been BCS at our institution. Therefore, in order to further reduce the number of mastectomies performed at our institution, we must evaluate the TMPP patient group to identify potential areas to de-escalate surgery.

To identify factors that could further de-escalate early-stage breast cancer surgery, we evaluated the patient and tumour characteristics of the TMPP patient group compared to the TMMN patient group and patients receiving BCS (Table 1). Similar to the findings of other studies, TM was associated with younger age [11]. As expected, patients in the TMMN group tended to have larger pre-operative tumour sizes. Additionally, they were more likely to be lymph node-positive and were more likely to have originally presented as a palpable mass, both likely a reflection of the larger tumour size. Figure 3 also supports this finding, as the proportion of BCS and TMPP shifts to TMMN with increasing tumour size. The pre-operative tumour size was larger than the postoperative tumour size, in part because the post-operative pathology size only includes the invasive component due to the challenges of reliably combining the invasive and DCIS components in the final pathological size. This also raises the possibility that there may be some overestimation by imaging, however, the surgeon would have planned to remove that area based on the pre-operative information. There were no statistical differences in tumour morphology between the three groups, suggesting no over-treatment of invasive cancers compared to DCIS. Overall, patient and tumour characteristics seem to be more similar in patients in the BCS and TMPP groups than in the TMMN group. However, there was a higher bilateral cancer rate in the TMPP than in the BCS group, and this was statistically significant. For this study, we included patients in the bilateral cancer rate if they presented with bilateral cancer or if they had previous contralateral cancer surgery in the past for a primary tumour, but not a recurrence. This finding indicates that patients may choose mastectomy rather than BCS for complex reasons that may not be captured by our data collection. Perhaps patients hope to reduce the number of breast procedures performed or for fear of recurrence. This may be reflected in the finding that our single operation rate (EUSOMA QI 9a and 9b) for both invasive cancers and DCIS decreased to below the target value when removing the TMMN group from the calculation. Interestingly, the TMPP group had a lower reconstruction rate than the TMMN group, suggesting that some may opt for mastectomy in order to avoid needing additional treatment, such as additional operations or radiotherapy. Some studies have found that proximity to a radiotherapy centre was a factor when deciding BCS or mastectomy [11]. Although most patients treated at our centre live locally, we do see patients from all health authorities around British Columbia, and we did not appreciate a geographical influence in the TMPP patient group. However, this analysis was limited because only postal codes were used, not actual proximity to the radiotherapy centre, which may not be updated or accurate and should be investigated further. Overall, the BCS and TMPP patient groups were similar but not the same. BCS rates may not represent the complexities in decision-making that cause patients to choose mastectomy over BCS, such as wanting the reduce the number of breast procedures performed, even when seemingly medically indicated.

Studies have identified increasing mastectomy rates despite the known safety and efficacy of BCS [6,7]. Multiple qualitative studies suggest that individual patient factors, including fear of recurrence, influence patient decision making [11,12,13,14]. Interestingly, Gu et al. [12] found that shared decision-making was associated with BCS, while mastectomy was associated with patient’s who perceived decision-making was largely carried out on their own. In another study performed at our institution evaluating average-risk women with unilateral breast cancer choosing contralateral prophylactic mastectomy (CPM), we found that adopting a united approach to educating patients and collaborating with other health professionals, such as nurses and plastic surgeons, about the use of CPM in this patient population resulted in a reduction in CPM rates [21]. While we used different inclusion and exclusion criteria for the present study, we also observed a decrease in CPM rates over time, although in this study we see that the BCS rates did not improve over time. The BCS rate for DCIS initially decreased and then increased again in 2017, although overall this was not statistically significant on trend analysis, however, the number of DCIS cases was smaller than the invasive. Other factors that may influence BCS rates in the future are the increasing indications for post-mastectomy radiotherapy [22,23] tailoring of patients for neoadjuvant chemotherapy, changing reconstructive options, and the introduction of oncoplastic surgery which facilitates BCS for larger tumours. Our results support the understanding that BCS rates are influenced by multiple factors and may not represent the full extent and complexities of decision-making in the surgical management of breast cancer [24].

While our study has strengths, including prospective practice data collection including a prospective collection of reasons for mastectomy, there are also some limitations. Our data did not collect information about patient ethnicity and reliably report patient’s geographic considerations, both of which would provide further insight into the decision making between BCS and TMPP. We also did not collect qualitative information from patients who chose mastectomies by patient preference and so could only make an assumption about patient-decision-making based on quantitative patient and tumour data. Collecting more detailed data about the decision-making behind BCS compared to TMPP could provide an avenue for future research to de-escalate surgical treatment of early-stage breast cancer.

As we worked on this study it became apparent that BCS rates are challenging to compare across jurisdictions without standardised inclusion and exclusion criteria. Exclusions vary between EUSOMA and American standards and both allude to cosmetic outcomes with tumour to breast ratio but do not indicate how to account for this. A study of BCS and mastectomy rates from the EUSOMA database [6] looked at post-operative pathology size and excluded patients with multifocal/multicentric, T3/T4, N2, or stage III disease. Additionally, we identified challenges in classifying and measuring tumours. Some reports described multiple areas and had more than one area biopsied and may be classified as multifocal (with individual measurements of each foci) and other tumours may have been measured across the whole abnormal area or had a single biopsy. For this reason, we opted to exclude patients coded as having multifocal disease, although these likely had areas larger than 3 cm so would not have been part of the EUSOMA QI calculation. Although it would be ideal to calculate BCS rates looking at all cases when we review the indication for surgery and reason for mastectomy we see the multitude of clinical factors that are considered. These details would not be available in administrative datasets that would be used for calculating QIs for larger regions such as different areas of a country or province but would be informative for institutions when wanting to review their own practice. In the literature review, we also see studies reporting post-operative pathology size, whereas the surgeon makes the decision based on the pre-operative size information. These considerations underscore the importance of surgeon collected practice data as advocated for by EUSOMA, ACS and the Canadian Partnership Against Cancer. Furthermore, it is challenging to calculate QIs based on size alone since it does not take into account proportion to the size of the breast, and this may vary across cultures and countries.

In terms of assessing whether we should apply European and American QIs, our finding that 80% of patients with a unifocal tumour would be eligible for BCS at a size of 2.5 cm, indicates that the European QI at a size of 3 cm may not be attainable in our multicultural patient population. Taking all of these factors into consideration raises the possibility of considering assessing BCS rates for T1 tumours in a multicultural population if we want to look at BCS QIs. On the other hand, CPM rates may offer a more actionable opportunity to de-escalate surgery for early-stage breast cancer than BCS rates.

## 5. Conclusions

To conclude, our institution found that our mastectomy rates were largely driven by medically necessary mastectomies. As well, our results highlighted the limitations of the QI for BCS rates in a multicultural population in that it cannot fully capture the extent of decision-making since tumour size does not take into account the relative proportion to the patient’s size of breast and ability to achieve clear margins and adequate cosmesis. In our patient population, 80% of tumours were eligible for BCS at a size of 2.5 cm.

## Figures and Tables

**Table 1:** Patient and tumour characteristics.

Heading		BCS (N = 1651)	TMMN (N = 387)	TMPP (N = 273)	*p*-Value		
					TMMN vs. BCS	TMPP vs. BCS	TMMN vs. TMPP
Patient Age (Continuous)	Mean	60.2	58.1	61.5	0.007	0.107	0.001
	Median	60	55	62	<0.001	0.131	<0.001
	Range	23–100	29–93	30–92	n/a	n/a	n/a
	n	1651	387	273	n/a	n/a	n/a
Patient Age (Categorical)	<40	47 (2.8%)	29 (7.5%)	8 (2.9%)	<0.001	0.001	0.042
	40 to 75	1464 (88.7%)	303 (78.3%)	223 (81.7%)			
	>75	140 (8.5%)	55 (14.2%)	42 (15.4%)			
	n	1651	387	273	n/a	n/a	n/a
CPM	Rate	n/a	68 (17.6%)	66 (24.2%)	n/a	n/a	0.042
	n	1651	387	273	n/a	n/a	n/a
Bilateral Cancer	Rate	31 (1.9%)	32 (8.3%)	25 (9.2%)	<0.001	<0.001	0.681
	n	1651	387	272	n/a	n/a	n/a
Reconstruction	Rate	n/a	211 (75.1%)	106 (39.0%)	n/a	n/a	<0.001
	n	1650	281	272	n/a	n/a	n/a
Presenting Problem	Mass	588 (37.4%)	249 (68.0%)	131 (49.6%)	<0.001	<0.001	<0.001
	Imaging Abnormality	958 (61.0%)	101 (27.6%)	121 (45.8%)			
	Nipple Discharge	10 (0.6%)	10 (2.7%)	7 (2.7%)			
	Breast Pain	6 (0.4%)	1 (0.3%)	1 (0.4%)			
	n	1571	366	264	n/a	n/a	n/a
Morphology	DCIS	326 (20.2%)	99 (26.4%)	48 (17.7%)	0.011	0.541	0.028
	IDC	1204 (74.8%)	252 (67.2%)	207 (76.4%)			
	Other (LCIS, Paget’s, ILC)	80 (5.0%)	24 (6.4%)	16 (5.9%)			
	n	1610	375	271	n/a	n/a	n/a
Pre-Op Lymph Node Status	Positive	62 (4.5%)	63 (19.3%)	17 (6.9%)	<0.001	0.164	<0.001
	n	1383	326	248	n/a	n/a	n/a
Post-Op Lymph Node Status	Positive	354 (28.6%)	149 (40.8%)	63 (25.3%)	<0.001	0.552	<0.001
	n	1237	365	249	n/a	n/a	n/a
Pre-op Tumour Size (mm)	Mean	17.5	42.2	20.7	<0.001	<0.001	<0.001
	n	1575	363	251	n/a	n/a	n/a
Post-op Tumour Size (mm)	Mean	17.8	29.5	17.7	<0.001	0.908	<0.001
	n	1527	358	243	n/a	n/a	n/a
ER	Positive	1073 (90.8%)	215 (85.0%)	174 (85.3%)	0.016	0.038	0.926
	n	1182	253	204	n/a	n/a	n/a
PR	Positive	971 (83.8%)	196 (78.1%)	146 (75.3%)	0.045	0.010	0.486
	n	1159	251	194	n/a	n/a	n/a
Her2	Positive	117 (10.6%)	37 (15.3%)	23 (12.0%)	0.059	0.577	0.317
	n	1107	242	192	n/a	n/a	n/a
LVI	Positive	233 (21.3%)	88 (38.6%)	39 (20.1%)	<0.001	0.699	<0.001
	n	1093	228	194	n/a	n/a	n/a

Patient and tumour characteristics for patients receiving BCS, a medically necessary total mastectomy (TMMN), or patient preference total mastectomy (TMPP) as their first breast cancer surgery. ER, PR, Her2 and LVI status are evaluated in only the subset of patients with invasive ductal cancer (IDC) and invasive lobular carcinoma (ILC).

**Table 2:** Six QIs from NAPBC and EUSOMA were compared for compliance.

Quality Indicator	Minimum Standard	Target	5-Year Rate (2013–2017)	Remove Medically Necessary TM
NAPBC Standard 2.3: Breast-conserving surgery is offered to appropriate patients with breast cancer. A target rate of at least 50 percent of all eligible patients diagnosed with early-stage breast cancer (Stage 0, I, II) is treated with breast-conserving surgery	n/a	50%	71.44%	81.25%
EUSOMA 9a: Proportion of patients (invasive cancers) who received a single (breast) operation for the primary tumour (excluding reconstruction)	80%	90%	88.80%	84.50%
EUSOMA 9b: Proportion of patients (DCIS only) who received just one operation (excluding reconstruction)	70%	90%	80.30%	69.64%
EUSOMA 9c: Proportion of patients receiving immediate reconstruction at the same time of mastectomy	40%	none	48.94%	28.90%
EUSOMA 11c: Proportion of patients (BRCA1 and BRCA2 patients excluded) with invasive breast cancer not greater than 3 cm (total size, including DCIS component) who underwent BCT as primary treatment	70%	85%	77.10%	83.38%
EUSOMA 11d: Proportion of patients with non-invasive breast cancer not greater than 2 cm who underwent BCT	80%	90%	84.90%	90.14%

Listed are the descriptions of each QI, the minimum standard, and the target rate. Our institutions 5-year rate was indicated including the TMMN patient group and without.

**Table 3:** Time and age trends for BCS rates by tumour type (invasive cancer or DCIS) and size (cm).

Predictor	Comparison vs. Reference	Tumour Type	Cut-Off	Odds Ratio	95% Confidence Interval		*p*-Value
					Lower	Upper	
Year of Operation	Each year later	DCIS	<2 cm	1.040	0.858	1.262	0.691
	Each year later	Invasive	<2 cm	1.066	0.964	1.178	0.213
	Each year later	Invasive	<3 cm	1.065	0.982	1.154	0.129
Age Category	40 to <75 vs. <40	DCIS	<2 cm	3.689	0.888	14.385	0.058
	75+ vs. <40	DCIS	<2 cm	2.933	0.476	20.358	0.250
	40 to <75 vs. <40	Invasive	<2 cm	3.145	1.571	6.221	0.001
	75+ vs. <40	Invasive	<2 cm	2.127	0.984	4.575	0.053
	40 to <75 vs. <40	Invasive	<3 cm	2.542	1.495	4.304	0.001
	75+ vs. <40	Invasive	<3 cm	1.806	0.996	3.272	0.050

## Data Availability

All data generated or analysed during this study are reported in this published article.
